# A web-based dynamic Nomogram for predicting instrumental activities of daily living disability in older adults: a nationally representative survey in China

**DOI:** 10.1186/s12877-021-02223-9

**Published:** 2021-05-17

**Authors:** Li Zhang, Huijie Cui, Qiuzhi Chen, Yan Li, Chunxia Yang, Yanfang Yang

**Affiliations:** Department of Epidemiology and Biostatistics, West China School of Public Health and West China Fourth Hospital, Sichuan University, No.17 Section 3, Renmin South Road, Chengdu, 610041 Sichuan China

**Keywords:** Instrumental activities of daily living disability, Dynamic nomogram, Prediction model

## Abstract

**Background:**

Instrumental Activities of Daily Living (IADL) disability is a common health burden in aging populations. The identification of high-risk individuals is essential for timely targeted interventions. Although predictors for IADL disability have been well described, studies constructing prediction tools for IADL disability among older adults were not adequately explored. Our study aims to develop and validate a web-based dynamic nomogram for individualized IADL disability prediction in older adults.

**Methods:**

Data were obtained from the China Health and Retirement Longitudinal Study (CHARLS). We included 4791 respondents aged 60 years and over, without IADL disability at baseline in the 2011 to 2013 cohort (training cohort) and 371 respondents in the 2013 to 2015 cohort (validation cohort). Here, we defined IADL disability as needing any help in any items of the Lawton and Brody’s scale. A web-based dynamic nomogram was built based on a logistic regression model in the training cohort. We validated the nomogram internally with 1000 bootstrap resamples and externally in the validation cohort. The discrimination and calibration ability of the nomogram was assessed using the concordance index (C-index) and calibration plots, respectively.

**Results:**

The nomogram incorporated ten predictors, including age, education level, social activity frequency, drinking frequency, smoking frequency, comorbidity condition, self-report health condition, gait speed, cognitive function, and depressive symptoms. The C-index values in the training and validation cohort were 0.715 (bootstrap-corrected C-index = 0.702) and 0.737, respectively. The internal and external calibration plots for predictions of IADL disability were in excellent agreement. An online web server was built (https://lilizhang.shinyapps.io/DynNomapp/) to facilitate the use of the nomogram.

**Conclusions:**

We developed a dynamic nomogram to evaluate the risk of IADL disability precisely and expediently. The application of this nomogram would be helpful for health care physicians in decision-making.

## Background

Functional disability is a common health problem in older adults. It causes multiple adverse events such as falls, hospitalization, and mortality [1–3] and places a heavy burden on health care systems [4]. Functional disability is defined as dependency in performing daily activities which are categorized into activities of daily living (ADL) and instrumental activities of daily living (IADL) [5]. ADL disability refers to needing help in routine self-care activities, whereas IADL disability is more related to living independently under a given circumstance [6, 7]. IADL disability in most of older adults often precedes ADL disability, following a hierarchical pattern, and is potentially easier to reverse [8, 9]. Therefore, identifying people at risk for IADL disability is critically essential for timely, targeted interventions to delay, slow, or even partially reverse the process of becoming care-dependent.

Predictors for IADL disability have been identified and well documented in many studies, including demographic characteristics, chronic conditions, health behaviors, and physical performance measures [10–13]. As a multifactorial health problem, IADL disability is best predicted by considering multiple domains of risk rather than any single risk factor [14]. However, studies that integrate multiple factors of IADL disability and construct prediction models are scarce. The existing prediction models have focused on ADL disability instead of IADL disability. A previous study developed a prediction model using the Frailty Index, but IADL disability was not the primary outcome and the model accuracy needs further improvement (C-index = 0.61) [15].IADL disability provides essential information for assessing whether an older person could be able to live independently in the community and has proved to be a more sensitive predictor of functional disability and mortality than ADL disability [16, 17]. Hence, the development and validation of the IADL prediction model are worth exploring.

Nomogram, as a prediction tool, has been widely applied to quantify the likelihood of specific events of interest. It can provide readily individualized risk estimation and deliver visualized results, thus facilitating decision-making by physicians and policy makers [18, 19]. Nevertheless, ordinary graphical nomograms may restrict their clinical applicability due to inconvenient risk calculations among non-statistical audiences [20, 21]. Therefore, we sought to develop and validate a web-based dynamic nomogram for IADL disability prediction among the Chinese older community population.

## Methods

### Study design and participants

Data were obtained from the China Health and Retirement Longitudinal Study (CHARLS), a nationally representative longitudinal survey among Chinese middle-aged and older adults. A wide range of information on socioeconomic status, health circumstances, and anthropometric and laboratory measurements were collected [22]. The CHARLS baseline survey was conducted from 2011 to 2012, involving 150 counties and 450 villages/resident committees in 28 provinces. Follow-up surveys were conducted in 2013, 2015, and 2018, respectively. Detailed descriptions of the survey design and procedures were available elsewhere.

**Fig. 1:**
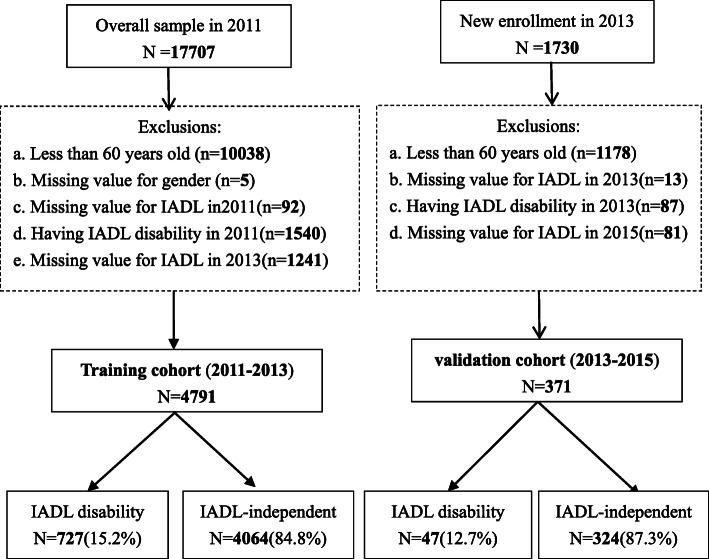
Study flow

In our study, we selected the respondents who participated in the baseline (2011 wave of CHARLS) as the training cohort, while the validation cohort consisted of the newly enrolled respondents in the 2013 wave. Incidence of IADL at two-year follow-up in each cohort was considered as the outcome of interest. The exclusion criteria were as follows: aged under 60 years old; had IADL disability at baseline; lacking key variables such as gender, age, or IADL status at baseline and follow-up. The detailed exclusion was shown in Fig. 1.

All the respondents signed informed consent at the time of participation and this study was approved by the Institutional Review Board of Peking University (IRB00001052–11014).

### Outcome

IADL disability was evaluated by Lawton and Brody’s scale, which refers to the abilities required for living independently in the community including doing housework, cooking, taking medicine, shopping, and taking care of finances [23]. Respondents were asked to choose from the four corresponding answers for each item: (1) No, I do not have any difficulty, (2) I have difficulty but still can do it, (3) Yes, I have difficulty and need help, and (4) I cannot do it. In our study, participants who reported needing any help in any items were classified as with IADL disability [5, 7].

### Candidate predictors

Candidate predictors were measured at baseline and consisted of sociodemographic factors, lifestyle variables, clinical factors, and physical performance measures. Sociodemographic factors included age, gender, marital status, and education level. Lifestyle variables included social activity frequency, drinking frequency, smoking frequency, and duration of night sleep. Clinical factors included body mass index (BMI), self-report health condition, comorbidity condition, depressive symptoms, and cognitive function. The physical performance measure was gait speed (m/s).

#### Sociodemographic factors

Age was categorized into four groups: 60–64, 65–69, 70–74, and older than 75 years [9]. Marital status included two categories: (a) married or cohabiting and (b) another marital status, including separated, divorced, widowed, and never married [24]. Furthermore, education levels were classified into illiterate, primary school and below, middle school, high/vocational school, and college and above [9].

#### Lifestyle variables

Social activity frequency was classified into the following four categories: never, not regularly, almost every week, and almost daily. Moreover, drinking frequency was classified into the following four categories: never, quit drinking, less than once a month, and more than once a month. Smoking frequency was divided into the following four categories, never, quit smoking, less than 20 cigarettes a day, and more than 20 cigarettes a day. Finally, the duration of night sleep was allocated into the following three categories: less than 7 h, 7 to 8 h, and more than 8 h [25].

#### Clinical factors

BMI was used with the following WHO cut-off points for Chinese: underweight (BMI < 18.5 kg/m^2^), normal weight (BMI = 18.5 kg/m^2^ to 24 kg/m^2^), and overweight or obese (BMI ≥ 24 kg/m^2^) [26]. Self-report health condition was classified into the following five categories: very good, good, fair, poor, and very poor. Suffering from two or more self-report chronic diseases was defined as comorbidity condition.

Depressive symptoms were assessed using the Center for Epidemiologic Studies Depression Scale-10 items (CES-D-10) [27], which has good sensitivity, specificity, and predictive characteristics [28, 29]. The CES-D10 contains 10 items with 4 response options, ranging from 0 (“rarely or none of the time”) to 3 (“most or all of the time”). A cutoff score ≥ 10 on the 0–30 CES-D-10 was optimal to identify respondents who had significant depressive symptoms [29, 30].

Cognitive function was calculated using two kinds of tests: episodic memory and mental intactness. Global cognitive scores were calculated as the sum of these two tests and ranged from 0 to 21 [31, 32]. In the episodic memory test, the participants were asked to memorize and recall ten words immediately (immediate recall) and several minutes later (delayed recall). The episodic memory score was the average score of the immediate recall and delayed recall tests and ranged from 0 to 10 [32]. The mental intactness test was based on selected questions from the mental status questions of the Telephone Interview of Cognitive Status (TICS) battery. In CHARLS, it included repeated subtraction by 7 s from 100, identifying the date, season, and day of the week, and a figure-drawing test. Answers to these questions were summed into a mental intactness score that ranged from 0 to 11 [31, 32].

#### Physical performance measures

In the test of gait speed, respondents were asked to walk along a straight 2.5-m flat course twice (there and back) at their usual speed. The average speed of the two trials was used in the analysis [31].

### Statistical analysis

Descriptive statistics were conducted to characterize the study populations. Continuous variables were expressed as median and quartile (non-normal distribution), and categorical variables were expressed as frequencies and proportions. Simple and multivariable logistic regression models were applied to estimate the relative risk (RR) and corresponding 95% confidential interval (CI) for every candidate predictor. Two-way interactions were tested and, if found, the interaction terms were included in a full model. The final multivariable logistic regression model selection was performed by a backward stepwise selection with the Akaike information criterion (AIC). Subsequently, we constructed a nomogram based on the multivariable analysis results of the final model, using the RMS package of R software.

To evaluate the nomogram’s performance, we used the internal validation via a bootstrap method with 1000 resamples and external validation in the validation cohort. The performance of the nomogram was evaluated by considering discrimination and calibration. Discrimination refers to the models’ ability to distinguish patients with different outcomes and takes the concordance index (C-index) as the measuring tool. The C-index ≥0.70 is the criterion of good discrimination [8]. Calibration refers to the consistency between the actual outcomes and predicted outcomes and was evaluated by calibration plots. The 45-degree line represented perfect calibration. If points were close to the 45-degree line, the calibration was better [33]. Finally, we used the “DynNom” package to construct a dynamic nomogram, which can dynamically predict the IADL disability on the website [21].

Statistical analyses were conducted with R software (version 3.0.2; http://www.Rproject.org) and SPSS (version 20.0). All statistical tests were two-sided, and *p* values of less than 0.05 were considered statistically significant.

## Results

### Baseline characteristics and incidence of IADL disability

The characteristics of participants in the training and validation cohorts are reported in Table 1. During the study period, 4791 were from the training cohort and 371 were in the validation cohort. Table 2 displays the incidence of IADL disability stratified by age and gender. There were 727(15.2%) and 47(12.7%) participants developing IADL disability in the training and validation cohort, respectively. The IADL disability incidence tended to be higher among female participants and older adults with advanced age in both cohorts.

**Table 1:** Baseline Characteristics in Training and Validation Cohorts

Variables	Cohorts, No. (%)	
	Training (4791)	Validation (371)
**Age (Years)**		
60 ~	2022 (42.2%)	143 (38.5%)
65 ~	1209 (25.2%)	103 (27.8%)
70 ~	849 (17.7%)	69 (18.6%)
75 ~	711 (14.8%)	56 (15.1%)
**Gender**		
Male	2508 (52.3%)	192 (51.8%)
Female	2283 (47.7%)	179 (48.2%)
**Education**		
Illiterate	2501 (52.2%)	209 (56.3%)
Primary school	1313 (27.4%)	79 (21.3%)
Middle school	617 (12.9%)	51 (13.7%)
High school	257 (5.4%)	27 (7.3%)
College and above	103 (2.2%)	5 (1.4%)
**Marital status**		
Married/Cohabitated	3873 (80.8%)	293 (79.0%)
Other	918 (19.2%)	78 (21.0%)
**Social activity**		
Never	2467 (51.5%)	146 (39.4%)
Not regularly	526 (11.0%)	46 (12.4%)
Almost Weekly	506 (10.6%)	39 (10.5%)
Almost daily	1292 (27.0%)	140 (37.7%)
**Smoking**		
Never	2947 (61.5%)	217 (58.5%)
Quit	596 (12.4%)	46 (12.4%)
Less than 20 /day	556 (11.6%)	58 (15.6%)
More than 20 /day	692 (14.4%)	50 (13.5%)
**Drinking**		
Never	2749 (57.4%)	202 (54.4%)
Quit	487 (10.2%)	37 (10.0%)
Less than once/month	334 (7.0%)	35 (9.4%)
More than once/month	1221 (25.5%)	97 (26.1%)
**duration of night sleep (hours)**		
7–8	1859 (38.8%)	137 (36.9%)
< 7	2541 (53.0%)	197 (53.0%)
> 8	391 (8.1%)	37 (9.9%)
**BMI**		
Normal	2675 (55.8%)	179 (48.2%)
Underweight	499 (10.4%)	30 (8.1%)
Overweight	1617 (33.8%)	162 (43.7%)
**Comorbidity**		
0	1355 (28.3%)	99 (26.7%)
1	1475 (30.8%)	100 (27.0%)
≥ 2	1961 (40.9%)	172 (46.4%)
**Self-report health conditions**		
Very good	127 (2.65%)	38 (10.2%)
Good	551 (11.5%)	62 (16.7%)
Fair	1572 (32.8%)	179 (48.2%)
Poor	1813 (37.8%)	82 (22.1%)
Very poor	728 (15.2%)	10 (2.70%)
**Depression**		
Normal	3086 (64.4%)	252 (67.9%)
Depression	1705 (35.6%)	119 (32.1%)
	Median(Q1-Q3)	Median(Q1-Q3)
**Cognitive function**	10.0 [6.50;13.50]	11.0 [7.00;13.50]
**Gait speed (m/s)**	0.64 [0.50;0.79]	0.68 [0.57;0.82]

**Table 2:** Incidence of IADL disability in the Training and Validation Cohorts

	Training Cohort		Validation Cohort	
Variables	IADL disability	IADL-Independent	IADL disability	IADL-Independent
**Age (years)**				
60 ~	212 (29.2%)	1810 (44.5%)	10 (21.3%)	133 (41.0%)
65 ~	159 (21.9%)	1050 (25.8%)	13 (27.7%)	90 (27.8%)
70 ~	165 (22.7%)	684 (16.8%)	8 (17.0%)	61 (18.8%)
75 ~	191 (26.3%)	520 (12.8%)	16 (34.0%)	40 (12.3%)
**Gender**				
Male	344 (47.3%)	2164 (53.2%)	23 (48.9%)	169 (52.2%)
Female	383 (52.7%)	1900 (46.8%)	24 (51.1%)	155 (47.8%)
**Overall**	727 (15.2%)	4064 (84.8%)	47 (12.7%)	324 (87.3%)

### Predictor variables and construction of models

In the univariable logistic regression analysis (Table 3), we identified all factors were associated with IADL disability except smoking frequency. Table 4 displayed the results of the full model and the final model. No significant pairwise interactions were found in our multivariable logistic regression model (data not shown). The final multivariable logistic regression model yielded the following ten variables: age, education level, social activity frequency, drinking frequency, smoking frequency, comorbidity condition, self-report health condition, gait speed, cognitive function, and depressive symptoms. We also compared the predictive performance of the full model and the final model in Table 4. These two models have almost similar C-index, and the *P* values of Hosmer–Lemeshow tests in two models were both > 0.05, showing they have almost the same predictive performances. Since the full model was too complex for routine use, the simplified final model was used in the subsequent analysis.

**Table 3:** Univariable logistic regression analysis of the Training Cohorts

Variables	RR(95% ***CI)***	***P*** value
**Age (years)**		<0.001
60 ~	1.000 (Reference)	
65 ~	1.293 (1.038 ~ 1.610)	0.022
70 ~	2.060 (1.650 ~ 2.571)	<0.001
75 ~	3.136 (2.520 ~ 3.902)	<0.001
**Education**		<0.001
Illiterate	1.000 (Reference)	
Primary school	0.622 (0.515 ~ 0.752)	<0.001
Middle school	0.445 (0.334 ~ 0.593)	<0.001
High school	0.261 (0.153 ~ 0.444)	<0.001
College and above	0.170 (0.062 ~ 0.465)	0.001
**Social activity**		<0.001
Never	1.000 (Reference)	
Not regularly	0.820 (0.631 ~ 1.066)	0.138
Almost Weekly	0.586 (0.436 ~ 0.789)	<0.001
Almost daily	0.671 (0.552 ~ 0.815)	<0.001
**Smoking**		0.075
Never	1.000 (Reference)	
Quit	1.225 (0.972 ~ 1.544)	0.086
Less than 20 /day	0.850 (0.652 ~ 1.107)	0.227
More than 20 /day	0.870 (0.685 ~ 1.105)	0.254
**Drinking**		<0.001
Never	1.000 (Reference)	
Quit	0.825 (0.630 ~ 1.082)	0.165
Less than once/month	0.603 (0.422 ~ 0.860)	0.005
More than once/month	0.667 (0.547 ~ 0.814)	<0.001
**Self-report health condition**		<0.001
Very good	1.000 (Reference)	
Good	1.297 (0.642 ~ 2.621)	0.468
Fair	1.522 (0.784 ~ 2.958)	0.215
Poor	2.413 (1.251 ~ 4.656)	0.009
Very poor	3.592 (1.842 ~ 7.006)	<0.001
**Comorbidity condition**		<0.001
0	1.000 (Reference)	
1	1.176 (0.945 ~ 1.463)	0.147
≥ 2	1.572 (1.289 ~ 1.919)	<0.001
**Gait speed**	0.238 (0.164 ~ 0.345)	<0.001
**Cognitive function**	0.883 (0.867 ~ 0.900)	<0.001
**Depression**		
Normal	1.000 (Reference)	
Depression	1.691 (1.379 ~ 2.075)	<0.001
**Marital status**		
Married/Cohabitated	1.000 (Reference)	
Other	1.353 (1.120 ~ 1.635)	0.002
**Gender**		
Male	1.000 (Reference)	
Female	1.268 (1.083 ~ 1.485)	0.003
**duration of night sleep (hours)**		0.001
7–8	1.000 (Reference)	
< 7	1.260 (1.062 ~ 1.495)	0.008
> 8	1.538 (1.155 ~ 2.048)	0.003
**BMI**		<0.001
Normal	1.000 (Reference)	
Underweight	1.221 (0.955 ~ 1.562)	0.112
Overweight	0.733 (0.612 ~ 0.878)	0.001

All analyses were conducted at a 5% significance level; *CI* confidence interval

**Table 4:** Multivariable logistic regression analysis of the full model and final model in the Training Cohorts

Variables	Full Model^**a**^		Final Model^**b**^	
	RR(95%***CI)***	***P*** value	RR(95%***CI)***	***P*** value
**Factors Selected**				
**Age (years)**				
60 ~	1.000 (Reference)		1.000 (Reference)	
65 ~	1.255 (1.001 ~ 1.574)	0.049	1.256 (1.002 ~ 1.574)	0.048
70 ~	1.789 (1.413 ~ 2.263)	<0.001	1.748 (1.414 ~ 2.251)	<0.001
75 ~	2.553 (1.991 ~ 3.274)	<0.001	2.509 (1.975 ~ 3.189)	<0.001
**Education**				
Illiterate	1.000 (Reference)		1.000 (Reference)	
Primary school	0.897 (0.724 ~ 1.112)	0.323	0.908 (0.734 ~ 1.122)	0.370
Middle school	0.822 (0.597 ~ 1.133)	0.231	0.825 (0.601 ~ 1.133)	0.235
High school	0.502 (0.286 ~ 0.881)	0.016	0.500 (0.286 ~ 0.875)	0.015
College and above	0.340 (0.121 ~ 0.954)	0.040	0.347 (0.124 ~ 0.972)	0.044
**Social activity**				
Never	1.000 (Reference)		1.000 (Reference)	
Not regularly	0.953 (0. 723 ~ 1.256)	0.734	0.946 (0.718 ~ 1.245)	0.692
Almost Weekly	0.726 (0.533 ~ 0.989)	0.043	0.723 (0.531 ~ 0.984)	0.039
Almost daily	0.786 (0.637 ~ 0.970)	0.025	0.768 (0.624 ~ 0.946)	0.013
**Smoking**				
Never	1.000 (Reference)		1.000 (Reference)	
Quit	1.630 (1.226 ~ 2.165)	0.001	1.666 (1.283 ~ 2.163)	<0.001
Less than 20 /day	1.167 (0.858 ~ 1.587)	0.324	1.201 (0.901 ~ 1.601)	0.211
More than 20 /day	1.500 (1.112 ~ 2.025)	0.008	1.541 (1.175 ~ 2.019)	0.002
**Drinking**				
Never	1.000 (Reference)		1.000 (Reference)	
Quit	0.724 (0.538 ~ 0.975)	0.033	0.737 (0.550 ~ 0.987)	0.040
Less than once/month	0.736 (0.505 ~ 1.072)	0.110	0.744 (0.512 ~ 1.082)	0.122
More than once/month	0.666 (0.526 ~ 0.842)	0.001	0.681 (0.542 ~ 0.855)	0.001
**Self-report health condition**				
Very good	1.000 (Reference)		1.000 (Reference)	
Good	1.206 (0.585 ~ 2.486)	0.612	1.222 (0.593 ~ 2.520)	0.586
Fair	1.305 (0.657 ~ 2.589)	0.447	1.350 (0.681 ~ 2.679)	0.390
Poor	1.805 (0.913 ~ 3.569)	0.089	1.857 (0.940 ~ 3.670)	0.075
Very poor	2.154 (1.070 ~ 4.338)	0.032	2.240 (1.113 ~ 4.507)	0.024
**Comorbidity condition**				
0	1.000 (Reference)		1.000 (Reference)	
1	1.112 (0.882 ~ 1.400)	0.369	1.100 (0.874 ~ 1.386)	0.416
≥ 2	1.428 (1.144 ~ 1.783)	0.002	1.183 (0.991 ~ 1.413)	0.063
**Gait speed**	0.493 (0.330 ~ 0.737)	0.001	0.510 (0.342 ~ 0.761)	0.001
**Cognitive function**	0.925 (0.904 ~ 0.948)	<0.001	0.925 (0.903 ~ 0.947)	<0.001
**Depression**				
Normal	1.000 (Reference)		1.000 (Reference)	
Depression	1.190 (0.992 ~ 1.428)	0.061	1.251 (1.001 ~ 1.563)	0.049
**Factors Not Selected**				
**Marital status**				
Married/Cohabitated	1.000 (Reference)		NA	NA
Other	0.861 (0.697 ~ 1.062)	0.162	NA	NA
**Gender**				
Male	1.000 (Reference)		NA	NA
Female	0.981 (0.776 ~ 1.240)	0.873	NA	NA
**duration of night sleep (hours)**				
7–8	1.000 (Reference)		NA	NA
< 7	1.031 (0.8597 ~ 1.237)	0.742	NA	NA
> 8	1.244 (0.919 ~ 1.684)	0.158	NA	NA
**BMI**				
Normal	1.000 (Reference)		NA	NA
Underweight	0.902 (0.695 ~ 1.171)	0.440	NA	NA
Overweight	0.836 (0.690 ~ 1.014)	0.069	NA	NA
**Prediction performance**	**Full Model**		**Final Model**	
**AIC**	3770.6		3766.0	
**C-index (95%CI)**	0.716 (0.697 ~ 0.736)		0.715 (0.695 ~ 0.734)	
**Hosmer-Lemeshow test**^**c**^	χ^2^ = 8.252 (*P* value =0.409)		χ^2^ = 5.019(*P* value =0.756)	

All analyses were conducted at a 5% significance level; *CI* confidence interval

^a^The full model incorporated fourteen predictors, including age, gender, marital status, education level, social activity frequency, drinking frequency, smoking frequency, duration of night sleep, BMI, self-report health condition, comorbidity condition, depressive symptoms, cognitive function, and gait speed

^b^The final model incorporated ten predictors, including age, education level, social activity frequency, drinking frequency, smoking frequency, self-report health condition, comorbidity condition, depressive symptoms, cognitive function, and gait speed

^c^The Hosmer–Lemeshow (H-L) test was used to examine the calibration. A *P* value > 0.05 was considered well-calibrated

### Development of a nomogram

**Fig. 2:**
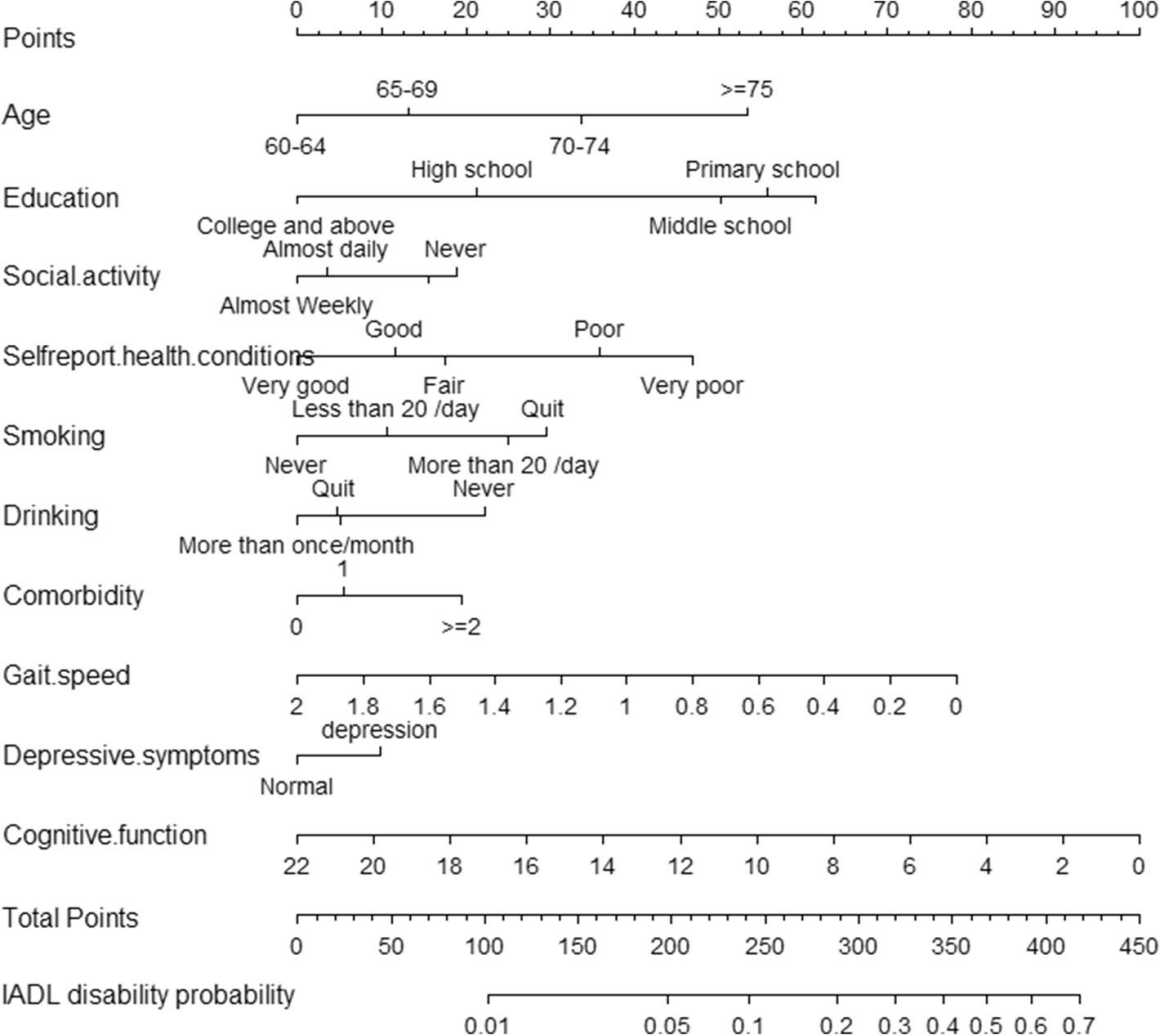
Nomogram for assessing the risk of IADL disability. To use the nomogram, an individual participant’s value is located on each variable axis, and a line is drawn upward to determine the number of points received for each variable value, add the points from all the variables, and draw a line from the total points axis to determine IADL disability probabilities at the lower line of the nomogram

The nomogram based on the final model is shown in Fig. 2**.** The risk of IADL disability can be calculated based on the sum of the assigned number of points for each factor in the nomogram. Higher total points were associated with a greater risk of IADL disability.

### Internal and external validation of a nomogram

**Fig. 3:**
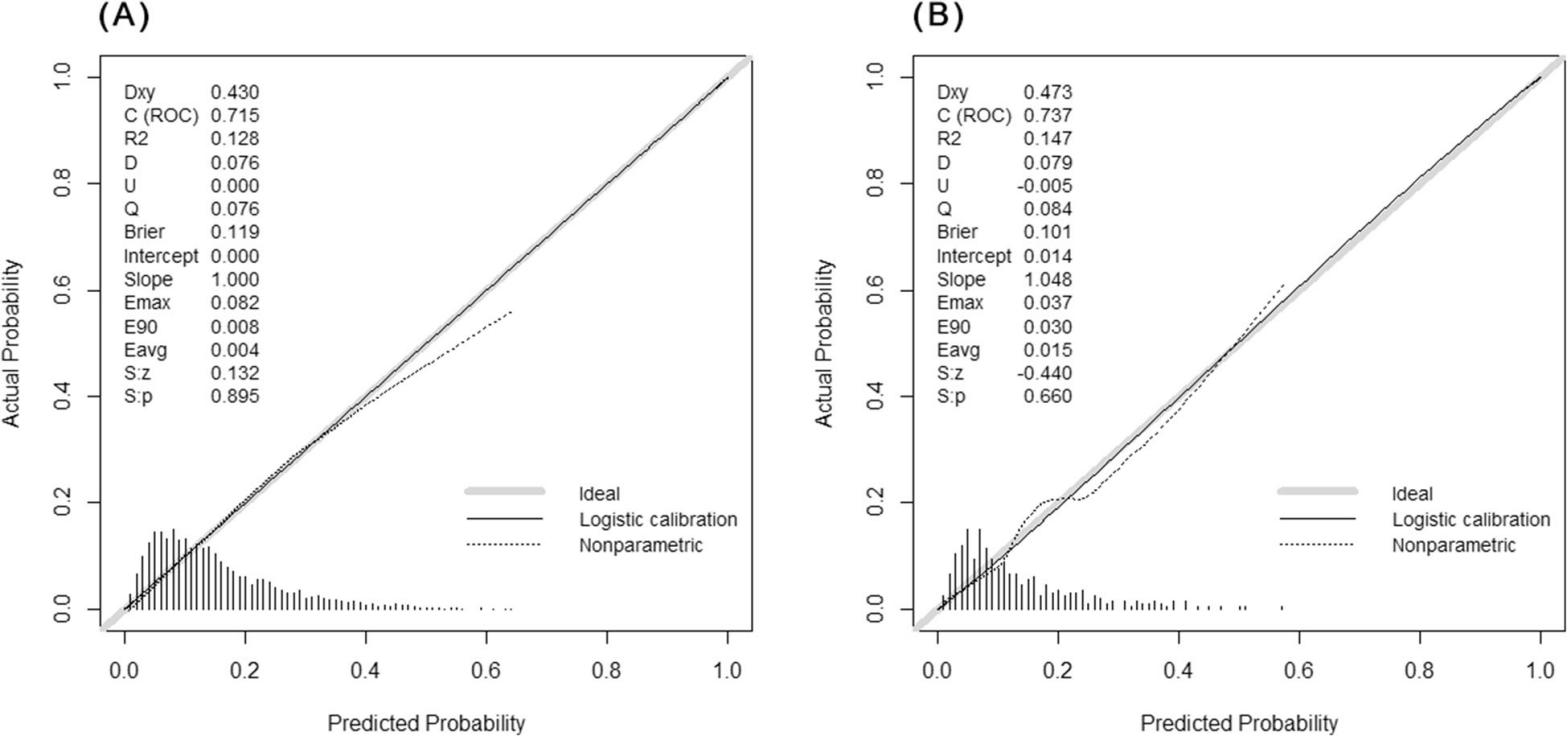
Calibration plots for the nomogram in both cohorts. **a** Training cohort. **b** Validation cohort. Nomogram-predicted probability and actual probability for IADL disability among participants with normal IADL at baseline were plotted in the x- and y-axis, respectively. The diagonal gray line represents an ideal plot for the calibration plot. The solid black line represents the performance of the nomogram, of which a closer match to the diagonal gray line indicates a better calibration

In the training cohort, the nomogram demonstrated good discrimination, with an unadjusted C index of 0.715 and a bootstrap-corrected C index of 0.702. The points of the calibration plot for the probability of IADL disability are close to the 45-degree line, showing good agreement between prediction by nomogram and actual observation (Fig. 3a). In the validation cohort, the nomogram displayed a C index of 0.737 with satisfactory discrimination for the IADL disability. There was also a good calibration for the risk estimation in the validation cohort (Fig. 3b).

### Web-based dynamic nomogram

**Fig. 4:**
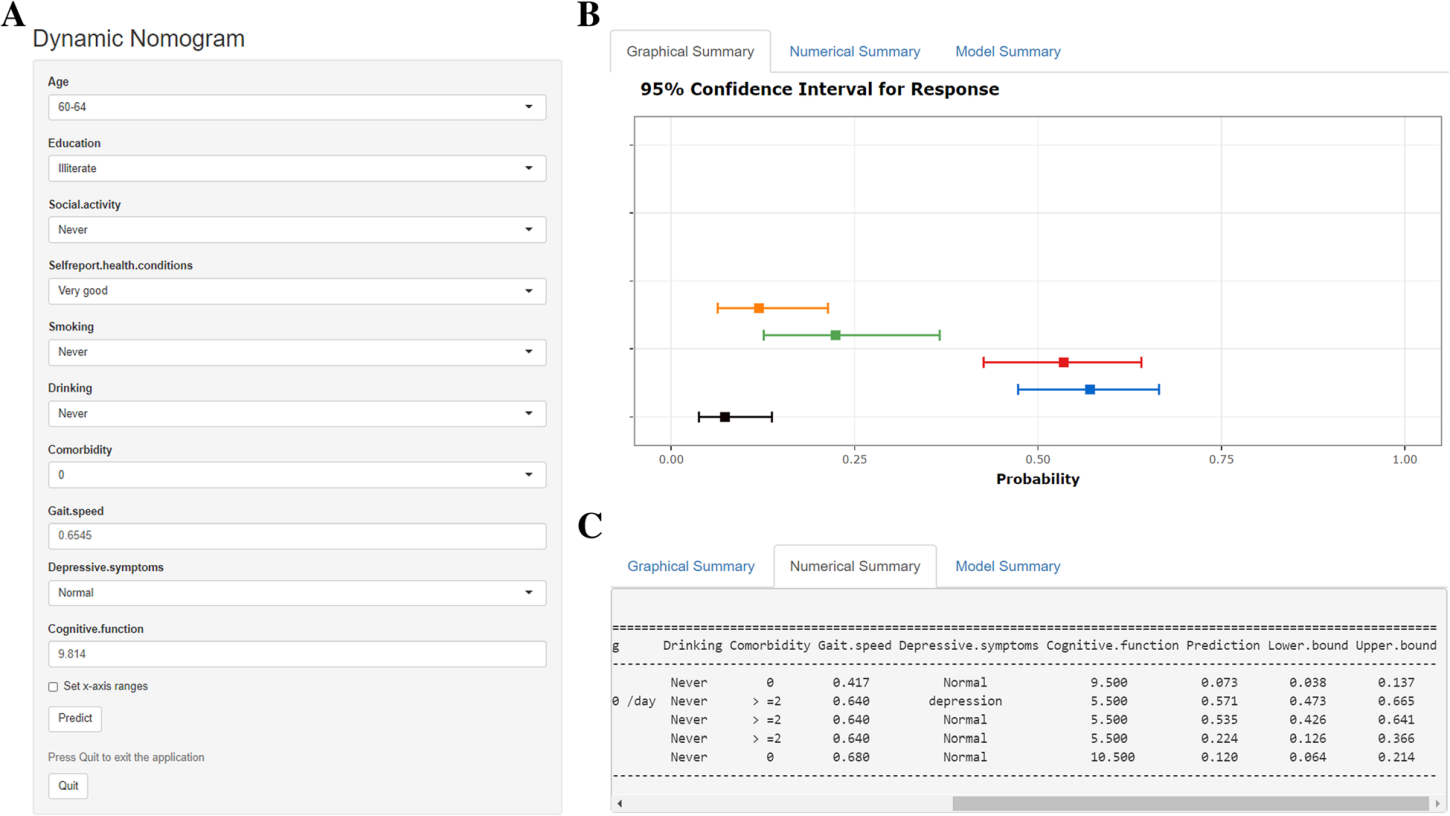
Web-based dynamic nomogram for predicting IADL disability. After entering a participant’s age, education level, social activity frequency, drinking frequency, smoking frequency, comorbidity condition, self-report health condition, gait speed, cognitive function, and depressive symptoms on https://lilizhang.shinyapps.io/DynNomapp/, we can get the participant’s corresponding probability of developing IADL disability. **a** Input interface: You can enter the related variables of participants in this interface. **b** Graphical summary: It represents participants’ corresponding probability and 95% confidence intervals of developing IADL disability. **c** Numerical summary: It shows the actual values of probability and 95% confidence intervals

We built a web-based calculator (https://lilizhang.shinyapps.io/DynNomapp/) to facilitate the use of the nomogram for clinicians. As shown in Fig. 4, the predicted probability of developing IADL disability can be easily obtained after inputting clinical variables and reading output results generated by the website.

## Discussion

To our knowledge, this study is the first attempt to establish a dynamic nomogram for predicting disability among Chinese older adults. We established an IADL disability prediction model integrating ten predictors including age, education level, social activity frequency, drinking frequency, smoking frequency, comorbidity condition, self-report health condition, gait speed, cognitive function, and depressive symptoms. Both internal validation and external validation demonstrated good discrimination and satisfactory calibration. The discriminative ability of our nomogram has a great improvement, from C-index of 0.61 in previously reported model to C-index of 0.715 in the current nomogram. Calibration plots in our study showed good agreement between prediction by nomogram and actual observation, which further verified the reliability of the nomogram.

Although extensive literature has identified predictors of IADL disability in community-living older adults, studies constructing prediction models for IADL disability are especially scarce, only with a few studies reporting on ADL disability prediction models. Nini H. Jonkman et al. developed and validated a clinical prediction model for ADL disability in three years of follow-up among older adults aged 65 ~ 75 years old. They selected 10 out of 22 predictors in the final prediction model including specific physical performance measures, age, body mass index, presence of depressive symptoms, and chronic conditions [34]. Kenneth E. Covinsky et al. constructed and validated an ADL disability prediction model among community-dwelling adults older than the age of 70. They included the following nine predictors in the final model: age, a comorbid condition, a measure of cognitive function, low BMI, and five measurements of functional limitation [14]. Three predictors including age, comorbid condition, and depressive symptoms in these two ADL disability models are also included in our model, suggesting that there might be shared underlying etiologies in ADL and IADL disability.

Our study supports previous studies that age is probably the most significant risk factor related to functional disability [35–37]. This could be explained by the nature of aging itself, in which the amount and quality of information needed for effective physical, intellectual, and social functioning are reduced [38]. In our study, comorbidity condition is independently associated with IADL disability in older populations. As chronic conditions become increasingly prevalent, research suggests comorbidity condition is much more highly associated with disability than single specific chronic condition [35]. Our study also aligns with current literature that older adults with depressive symptoms or cognitive impairment had a higher risk of IADL disability. The ability to engage in IADL requires higher cognitive function and more positive mood than ADL since IADL deals with more complex tasks [16, 39]. Development of strategies to ameliorate the depressive symptoms and delay the age-related cognitive decline may have a subsequent benefit for IADL ability [11]. Social activity participation is an important modifiable factor that could reduce risk of IADL disability and there is evidence that social network reduces the risk of depressive symptoms and boosts a sense of belonging and security among older adults [40, 41]. Some physiological mechanisms may explain these associations: specifically, a sound social participation integration has been linked to better immunologic, neuroendocrine, and cardiovascular functioning [40].

Previous studies have indicated that poor physical performance measures were associated with increased odds for IADL disability [13, 42, 43]. Gait speed, one of the most important physical performance measures, was recommended as an objective and reliable tool for predicting disability risk. Accumulating evidence has revealed that assessing gait speed alone is almost as good as performing the full battery of performance tests for the prediction of disability [44, 45]. For simplifying the model and improving the applicability in practice, we chose the gait speed alone to assess the association between the poor physical performance measures and IADL disability. Similarly, our study also validated this association. Based on these, older people should try to stay as active as possible to maintain favorable physical performance.

One interesting finding is that the IADL disability incidence was higher among female participants, but gender was not independently associated with IADL disability in the final model. Findings on IADL disability in older adults showed conflicting results, with some studies indicating greater IADL disability in older females and others found no gender difference [38]. Further relevant research is needed. Besides, in our study, participants who drink more than once a month had a lower risk of IADL disability than nondrinkers. Also, a previous study revealed that older adults who had small to moderate amounts of alcohol consumption were more likely to maintain functional status compared with nondrinkers [11, 35]. However, the details about the quantity and type of alcohol consumption require further research.

Early identification and prevention of disability should be a priority for healthy aging. Available evidence suggests that one-size-fits-all preventive interventions for IADL disability are unsuccessful because of the heterogeneity of older adults [46]. The application of a nomogram provides the possibility of individualized identification for older adults. Clinicians could take targeted interventions according to the scores of different items on nomogram for each subject, improving the efficiency of interventions. However, non-statistical audiences may find ordinary nomogram inconvenient because it requires risk calculation. In light of these considerations, we constructed a web-based dynamic nomogram (https://lilizhang.shinyapps.io/DynNomapp/) based on the logistic regression analysis. Health professionals could access the website directly on the mobile or computer anytime and anywhere and input corresponding predictors to obtain an individual’s IADL disability probability with 95% CI. This would undoubtedly simplify the application process and facilitate decision-making. Furthermore, it’s convenient for older adults and their caregivers to take targeted interventions according to the results of online nomogram. This strategy, combining the prediction model with the use of information and communication technology, considerably optimizes clinical application and promotes healthy aging.

Several limitations need to be mentioned in this study. First, IADL disability is sometimes a reversible event. Thus, some participants with IADL independence at the 2-year follow-up may have a prior short period of IADL disability. Similarly, some participants with IADL disability at 2 years may have subsequently recovered [14]. Secondly, although our nomogram incorporated an extensive range of predictors for IADL disability, some potential risk factors, such as social support, are currently not available in the study [47]. Therefore, to improve the efficiency of the nomogram, further research is warranted. Despite these limitations, this study established an intelligent, accurate, and convenient prediction tool for IADL disability in the older community population. We do not suggest replacing health professionals’ judgments with the dynamic nomogram, but rather believe it will inform and reinforce these judgments.

In conclusion, we have developed a pragmatic and personalized prediction tool for IADL disability among Chinese older adults. The nomogram retained its accuracy in an independent sample, demonstrating good discrimination and satisfactory calibration. The application of this nomogram will better help health care physicians quantify the risk of older individuals developing IADL disability and propose individualized intervention strategies.

## Data Availability

The datasets generated and analyzed during the current study are available in the CHARLS website, available in http://charls.pku.edu.cn/en.

## Abbreviations

ADL: Activities of daily living
IADL: Instrumental activities of daily living
CHARLS: China health and retirement longitudinal study
BMI: Body mass index
CES-D-10: Center for epidemiologic studies depression scale-10 items
TICS: Telephone interview of cognitive status
RR: Relative risk
95%CI: 95% confidential interval
AIC: Akaike information criterion
C index: Concordance index
